# The relationship between fibromyalgia and depression, anxiety, anxiety sensitivity, fear avoidance beliefs, and quality of life in female patients

**DOI:** 10.1097/MD.0000000000030868

**Published:** 2022-09-30

**Authors:** Sera Cetingok, Oznur Seker, Halil Cetingok

**Affiliations:** a Faculty of Health Science, Gerontology, Istanbul University – Cerrahpasa, Istanbul, Turkey; b Limit Educational Institutions, Istanbul, Turkey; c Division of Pain Medicine, Department of Anesthesiology, Istanbul Faculty of Medicine, Istanbul University, Istanbul, Turkey.

**Keywords:** anxiety, anxiety sensitivity, depression, fear avoidance beliefs, fibromyalgia, quality of life

## Abstract

The study aims to determine whether there is a relationship between fibromyalgia (FM) disease and depression, anxiety, anxiety sensitivity, fear-avoidance beliefs, and quality of life in female patients with a diagnosis of fibromyalgia. 37 female patients followed up with FM diagnosis in pain medicine clinic and a control group consisting of 37 healthy women were included in the study. Sociodemographic and Clinical Characteristics Data Form, Quality of Life Form, fear-avoidance beliefs questionnaire, Anxiety Sensitivity Index-3, Beck Anxiety Inventory, Beck Depression Inventory, and Visual Analogue Scale was applied to the participants. When the patients in the FM group were compared to the control group; Statistically lower scores in all Quality of Life Form subscales except emotional role difficulty and social functionality scores; statistically higher scores in both physical and work activity subscales in fear-avoidance beliefs questionnaire; statistically higher scores in cognitive symptoms subscale in Anxiety Sensitivity Index-3, Beck Anxiety Inventory, Beck Depression Inventory, and Visual Analogue Scale scores were found. In FM patients, it has been determined that anxiety, depression and perceived pain severity reduce social functionality and quality of life in areas such as mental health, physical function, and emotional role difficulties. It was determined that the functionality and quality of life of patients diagnosed with FM decreased in daily life. An important contribution of the study to the literature is that it shows that the behavior of avoiding activity due to pain-induced fear exacerbates the pain and even contributes to its chronicity. These results, which show the effects of anxiety, depression, anxiety sensitivity, and fear-avoidance behavior on the prognosis of the disease in FM patients, indicate that psychiatric evaluation and treatment in FM patients is an important factor that determines the functionality and quality of life.

## 1. Introduction

Fibromyalgia (FM) is a common and devastating syndrome that causes pain in the musculoskeletal system, sleep disturbance, fatigue, psychiatric disorders, and social dysfunction.^[1]^ FM often coexists with other chronic pain conditions with overlapping clinical features: chronic fatigue syndrome, irritable bowel syndrome, tension-type headache, migraine, temporomandibular disorder, multiple chemical sensitivity, restless leg syndrome, primary dysmenorrhea, interstitial cystitis, post-traumatic stress disorder, myofascial pain syndrome. The terminology of “central sensitivity syndromes” or “chronic overlapping pain conditions” describes this group of conditions.^[2]^

In addition to physical complaints such as pain, FM patients have also been shown to experience cognitive dysfunctions such as attention problems, problems in planning, difficulty in remembering, concentration difficulties, decreased vocabulary, poor verbal fluency, mental slowness, difficulty in thinking and making decisions.^[3]^

FM syndrome also has many emotional and affective effects. Fibromyalgia syndrome patients tend to experience high levels of stress, anger, and pain catastrophizing and these also contribute to the worsening of existing symptoms.^[3]^ Moreover, it has been shown that a significant portion of FM patients has various psychiatric comorbidities such as depression, panic disorder, and anxiety disorder.^[1]^ These cognitive and psychiatric aspects may have a predictive value on somatic symptoms such as pain.

In this study, an answer was sought to the question of whether there was a difference in the quality of life, fear-avoidance beliefs, anxiety, anxiety sensitivity (AS), and depression between female patients with FM who were followed up with the complaint of widespread pain and the control group without FM diagnosis.

## 2. Method

The study was carried out by cross-sectional examination of the patients followed up with the diagnosis of FM in the Pain Medicine Clinic of the Research Hospital, with the permission (dated 11.07.2017 and numbered 2017-593) from the local clinical research ethics committee. 37 female patients with FM and age-sex matched 37 healthy women participated in the study.

Inclusion criteria for the patient group were determined as 18 to 70 years old, female, being treated with FM diagnosis, voluntarily agreeing to participate in the study, and being literate.

Exclusion criteria for the patient group were determined as being under the age of 18 and over the age of 70, male, not in the FM diagnosis spectrum, illiteracy, mental retardation, alcohol, and substance use disorder.

Sociodemographic and Clinical Characteristics Data Form, Anxiety Sensitivity Index-3 (ASI-3), Quality of Life Form (SF-36), Visual Analogue Scale (VAS), Fear-Avoiding Beliefs Questionnaire (FABQ), Beck Depression Inventory, and Beck Anxiety Scales (BAI) were applied.

### 2.1. Sociodemographic and clinical information form

In the sociodemographic information form, the patients and the control group included age, gender, education status, and habitual status.

### 2.2. Quality of Life Form

The SF 36 questionnaire is a tool consisting of 36 items and 8 sub-titles that evaluate physical and social functions and mental health. These 8 sections are Physical Function, Physical Role Difficulty, Emotional Role Difficulty, Energy, Mental Health, Social Functioning, Pain, and General health. Subscales evaluate health between 0 and 100, with 0 indicating poor health and 100 indicating good health. It was developed by Ware et al.^[4]^

### 2.3. Fear Avoidance Beliefs Questionnaire

The FABQ, a 16-question questionnaire created by Waddel et al,^[5]^ has two subheadings: physical activity and work activity. Its main purpose is to show the effect of activity-induced fear and avoidance belief on pain and disability.

### 2.4. Anxiety Sensitivity Index-3

Fear of an event or situation is defined as AS to avoidance motive. ASI-3, the most commonly used scale to evaluate AS, which consists of three subscales (physical, cognitive, and social) and a total of 18 items, was first developed by Reiss and McNally.^[6]^

### 2.5. Beck Anxiety Inventory

BAI was developed by Beck et al^[7]^ to measure students’ anxiety levels. It is a Likert-type assessment tool in which scores ranging from 0 to 3 are given on the 21-item scale. A high total score indicates an increase in anxiety severity.

### 2.6. Beck Depression Inventory

Beck Depression Scale, which was developed by Beck et al, is used to diagnose depression; it has been used both to measure its severity and to follow treatment-related changes.^[8]^ On the scale consisting of 21 items, mood, pessimism, sense of failure, dissatisfaction, guilt, punishment, self-hatred, self-blame, desire to punish oneself, crying spells, irritability, social introversion drowsiness, indecisiveness, bodily image, impaired ability to work, sleep disorders, fatigue, fatigue, decreased appetite, weight loss, somatic complaints, and loss of sex drive. A high total score indicates an increase in the severity of depression.

### 2.7. Visual Analogue Scale

It is a valid and reliable scale that measures the severity of pain in one dimension. Pain severity definitions are written on both ends of a 10 cm line and the patient indicates where the pain severity status is appropriate on this line with a sign. For pain at one end, “I have no pain,” at the other end, very “severe pain” is written and the patient marks his current condition on this line. According to the VAS, I have no pain 0 points, severe pain is graded as 10 points.

### 2.8. Statistical method

While evaluating the findings obtained in the study, SPSS (IBM, New york, NY) 24 for Mac was used for statistical analysis. Descriptive statistical methods (mean, standard deviation, and frequency) were used while evaluating the study data. The chi-square test was used to compare qualitative data, and the Fisher exact chi-square test was used in case the expected frequencies were not met. The conformity of the data to the normal distribution was evaluated with the Kolmogorov–Smirnov test. The Student’s *t* test was used in the comparisons between two groups of the variables in which the parametric assumptions were met, and the Mann–Whitney *U* test was used in cases where the parametric assumptions were not met. In the study, Spearman’s rho was used for the variables that did not show the normal distribution in the analysis of qualitative and quantitative relationships. Although the significance was evaluated at *P* < .05 and *P* < .01 levels, Bonferroni correction was used where necessary and the significance values were determined according to this correction.

### 2.9. Sample size calculation

The sample size of this study was determined based on a previous study,^[9]^ according to power analysis the statistical power of the analysis was 0.81, the minimum required sample size for the study was 20 subject with a given an alpha level of 0.05, an anticipated effect size of 1.17 (large), and a desired statistical power level of 0.80.

## 3. Results

According to Table 1, no significant difference was found between the control and patient groups in terms of age, gender, educational status, and habitual status.

**Table 1:** Comparison of sociodemographic characteristics.

N = 74		Control group (n = 37)	Patient group (n = 37)	*T*	*P*
		Mean ± SD (median)	Mean ± SD (median)		
Age		45.08 ± 10.53 (43)	45.97 ± 9.95 (46)	−0.374	.709
		**N (%**)	**N (%**)	**χ^2^**	***P***
Sex†	Woman	37 (100)	37 (100)		1.000
	Man	–	–		
Education status†	Primary	14 (37.8)	19 (51.4)	1.502	.682
	Middle	9 (24.3)	8 (21.6)		
	High	8 (21.6)	6 (16.2)		
	University	6 (16.2)	4 (10.8)		
Habit†	None	27 (73.0)	31 (83.8)	1.806	.398
	Smoking	9 (24.3)	6 (16.2)		
	Alcohol	1 (2.7)	0 (0.0)		

SD = standard deviation.

Student *t* test,

† Fisher exact chi-square,

*P < .05 (mean ± SD).

According to Table 2, the scores of the patient group in all SF 36 subscales, except for emotional role difficulties and social functionality scores, were statistically significantly lower than the control group (*P* < .006).

**Table 2:** Comparison of 36-item short form survey results.

N = 74	Mean ± SD (median)		*U*/*t*	*P*
	Control group (n = 37)	Patient group (n = 37)		
Physical functioning	75.54 ± 24.26 (85)	47.56 ± 21.59 (50)	267.00	<.001**
Role physical	65.54 ± 39.68 (75)	13.51 ± 31.50 (0)	225.00	<.001**
Role emotional	56.75 ± 35.88 (67)	33.33 ± 44.44 (0)	457.50	.010
Energy/fatigue	50.00 ± 20.44 (45)	24.86 ± 23.87 (15)	312.50	<.001**
Emotional well-being	66.92 ± 15.39 (64)	46.48 ± 21.55 (48)	323.00	<.001**
Social functioning	68.24 ± 19.84 (75)	50.00 ± 30.47 (50)	443.00	.008
Bodily pain	71.95 ± 23.99 (78)	32.43 ± 25.00 (30)	180.50	<.001**
General health perceptions†	59.05 ± 17.43 (60)	31.75 ± 20.18 (35)	6.226	<.001**

SD = standard deviation.

Mann–Whitney *U* test,

† Student *t* test

** *P* < .006 [*P* value with Bonferroni correction (.05/number of comparisons; .05/8)] (mean ± SD).

According to Table 3, the scores of the patient group on both physical and division of labor subscales were statistically significantly higher than the control group (*P* < .025).

**Table 3:** Comparison of fear avoidance belief questionnaire results.

N = 74	Mean ± SD (median)		*U*	*P*
	Control group (n = 37)	Patient group (n = 37)		
Physical activity	7.92 ± 9.58 (4)	21.26 ± 8.80 (22)	213.00	<.001**
Work activity	3.64 ± 6.16 (0)	24.73 ± 17.95 (27)	221.50	<.001**

SD = standard deviation.

Mann–Whitney *U* test

** *P* < .025 [*P* value with Bonferroni correction (.05/number of comparisons; .05/2)] (mean ± SD).

According to Table 4, the ASI-3 Cognitive Symptoms scores of the patient group were statistically significantly higher than the control group (*P* < .016).

**Table 4:** Comparison of Anxiety Sensitivity Index-3 results.

N = 74	Mean ± SD (median)		*U*	*P*
	Control group (n = 37)	Patient group (n = 37)		
Physical concerns	17.37 ± 11.05 (17)	17.78 ± 8.53 (19)	615.50	.455
Social concerns	15.18 ± 6.31 (13)	20.48 ± 8.78 (19)	429.50	.006**
Cognitive concerns	10.27 ± 4.48 (9)	13.24 ± 6.20 (12)	496.00	.041

SD = standard deviation.

Mann–Whitney *U* test,

** *P* < .016 [*P* value with Bonferroni correction (.05/number of comparisons .05/3)] (mean ± SD).

According to Table 5, the Beck Anxiety Scale scores of the patient group were statistically significantly higher than the control group. (*P* < .05). Beck Depression Scale scores of the patient group were statistically significantly higher than the control group (*P* < .05). The VAS scores of the patient group were statistically significantly higher than the control group (*P* < .05).

**Table 5:** Comparison of BAI, BDI, and VAS results.

N = 74	Mean ± SD (median)		*U*	*P*
	Control group (n = 37)	Patient group (n = 37)		
BAI	11.70 ± 8.91 (10)	24.56 ± 14.70 (20)	296.50	<.001**
BDI	9.78 ± 9.38 (8)	21.13 ± 10.55 (19)	240.00	<.001**
VAS	2.54 ± 2.57 (2)	7.37 ± 1.87 (7)	111.00	<.001**

BAI = Beck Anxiety Inventory, BDI = Beck Depression Inventory, SD = standard deviation, VAS = Visual Analogue Scale.

Mann–Whitney *U* test

** *P* < .01 (mean ± SD).

According to Table 6, FABQ Work Division scores of FM patients show a positive, statistically significant relationship with VAS scores (*P* < .05). There is no statistically significant relationship between other variables.

**Table 6:** The relationship between FABQ and ASI-3, BAI, BDI, VAS in fibromyalgia patients.

		FABQ physical	FABQ work
ASI-3 physical concerns	r	0.243	0.296
	p	0.147	0.09
ASI-3 cognitive concerns	r	0.236	−0.033
	p	0.16	0.853
ASI-3 social concerns	r	0.229	0.127
	p	0.173	0.473
BAI	r	0.006	0.061
	p	0.97	0.731
BDI	r	0.201	0.152
	p	0.234	0.39
VAS	r	0.204	0.430*
	p	0.226	0.011

ASI-3 = Anxiety Sensitivity Index-3, BAI = Beck Anxiety Inventory, BDI = Beck Depression Inventory, FABQ = fear avoidance belief questionnaire, r = correlation coefficient, VAS = Visual Analogue Scale.

Spearman’s rho correlation,

* *P* < .05.

According to Table 7, ASI-3 physical symptoms scores of FM patients show a statistically significant positive correlation with Beck Depression (*P* < .01) and Beck Anxiety (*P* < .05) scores. ASI-3 Cognitive symptoms scores show a statistically significant positive correlation with Beck Depression and Beck Anxiety scores. (*P* < .01). ASI-3 social symptoms scores show a statistically significant positive correlation with Beck Depression (*P* < .01) and Beck Anxiety (*P* < .05) scores.

**Table 7:** The relationship of ASI-3 with BAI, BDI, VAS in fibromyalgia patients.

		ASI-3 physical concerns	ASI-3 cognitive concerns	ASI-3 social concerns
BAI	r	0.365*	0.424**	0.345*
	p	0.026	0.009	0.036
BDI	r	0.463**	0.573**	0.531**
	p	0.004	<0.001	0.001
VAS	r	0.261	0.114	0.036
	p	0.118	0.503	0.834

ASI-3 = Anxiety Sensitivity Index-3, BAI = Beck Anxiety Inventory, BDI = Beck Depression Inventory, r = correlation coefficient, VAS = Visual Analogue Scale.

Spearman’s rho correlation,

* *P* < .05,

** *P* < .01.

According to Table 8, ASI-3 physical symptoms scores of the control group show a statistically significant positive correlation with VAS scores (*P* < .01). ASI-3 Cognitive symptoms scores show a statistically significant positive correlation with Beck Anxiety (*P* < .05) Beck Depression and VAS scores (*P* < .01). ASI-3 social symptoms scores show a statistically significant positive correlation with Beck Depression and VAS scores (*P* < .01).

**Table 8:** The relationship of ASI-3 with BAI, BDI, VAS in control group.

		ASI-3 physical concerns	ASI-3 cognitive concerns	ASI-3 social concerns
BAI	r	−0.008	0.400*	0.306
	p	0.964	0.014	0.066
BDI	r	0.305	0.748**	0.490**
	p	0.066	<0.001	0.002
VAS	r	0.495**	0.465**	0.472**
	p	0.002	0.004	0.003

ASI-3 = Anxiety Sensitivity Index-3, BAI = Beck Anxiety Inventory, BDI = Beck Depression Inventory, r = correlation coefficient, VAS = Visual Analogue Scale.

Spearman’s rho correlation,

* *P* < .05,

** *P* < .01

## 4. Discussion

FM is a chronic pain syndrome that affects approximately 2% to 3% of the general population^[10]^ and 90% of the FM patients are women.^[11]^ The cause of FM is not clear.^[12]^ However, it is generally estimated that dysregulation in central sensory activity.^[13]^ There is no objective imaging or laboratory test for its diagnosis, and the diagnosis is generally made according to clinical markers.^[13]^ Although the main symptom is chronic widespread pain, it contains many symptoms accompanying the pain. Among them, fatigue, weakness, forgetfulness, morning stiffness, sleep disturbance, and some psychological comorbidities can be counted.^[14]^ As a result, FM significantly affects the quality of life negatively.^[15]^ However, evaluating FM, which has many physical and psychological components other than pain, by focusing only on the locomotor system and reducing it to pain only means denying the multidimensionality of the disease. FM is a disease in which psychological effects have an important place in the prognosis. Our study, it evaluated whether there was a significant difference between the FM patients and the control group in terms of the scales used and if there was a correlation in the sub-parameters of the scales in FM patients to investigate this relationship.

The SF 36 is a tool that evaluates physical functions, social functions, mental health, and consists of 8 sub-titles.^[4]^ These sub-headings are Physical Function, Physical Role Difficulty, Emotional Role Difficulty, Energy, Mental Health, Social Functioning, Pain, and General Health. Picavet et al^[16]^ evaluated patients with many different diseases that cause chronic pain in the Dutch population with SF 36. It was observed that FM patients got the lowest score in terms of all parameters except physical role difficulty, compared with the others.^[16]^ In our study, FM patients scored significantly lower than the control group in 6 sub-headings, except for emotional role difficulties and social functionality. In terms of emotional role difficulty and social functionality, *P* values were found to be .010 and .008, respectively, and the results were very close to the Bonferroni-corrected statistical significance limit (*P* < .006). Buskila et al^[17]^ in their study in which patients with FM were divided into two groups according to their gender, found that male patients had lower SF-36 scores than female patients. Although this result is less common than in female patients, it is consistent with the fact that male FM patients have a severe clinical condition, higher VAS scores, and a worse quality of life than females, which is consistent with being more resistant to treatment.^[18]^ In our study, the fact that all of the individuals in the patient and control groups were women, constitutes a limitation in this respect.

FABQ is created by Waddel et al^[5]^ on activity-induced fear and avoidance behavior on low back pain and chronic disability and was then tested in terms of different pain syndromes. Tezcan et al^[19]^ thought that exaggerated cognitive pain response caused fear and anxiety in patients and this would have reflections on behavior. With this prediction, they obtained similar results in their research in which they evaluated whether there was a difference between different chronic pain syndromes such as rheumatoid arthritis, hand osteoarthritis, and FM. FM, is a chronic pain syndrome that causes serious decreases in quality of life. According to the results of our study, in terms of both physical activity and work activity, the patients had significantly higher scores in FABQ than the healthy group without FM. The behavior of avoiding activity due to the fear induced by the pain exacerbates the pain and even contributes to its chronicity.^[20]^ Therefore, according to Turk and Wilson, fear and avoidance behavior should be questioned in the evaluation of patients with pain, and these behaviors should be recognized, confronted, and corrected as well as biological factors in the treatment of pain.^[20]^

In the study by Singh et al^[21]^ anxiety was found to be 87.5% and 23.6%, and depression 72.5% and 5%, respectively, between FM patients and the control group. This study indicates that anxiety and depression occur more frequently in FM patients. Pernambuco et al^[22]^ also found that the frequency of anxiety and depression was statistically significantly higher in FM patients. FM symptomatology does not only include pain, but also includes symptoms such as weakness, sleep disturbance, and forgetfulness. From this point of view, comorbid psychological conditions such as anxiety and depression that may affect all these symptoms may cause more severe FM clinic and symptoms in general, and therefore they should be considered in the evaluation of patients. ASI-3, BAI, and Beck Depression Inventory scores, which were found to be higher in FM patients in our study compared to the control group, support the aforementioned literature. In general, the opinion in the literature that the severity of pain in patients with FM is directly related to the anxiety and depression of the patient is predominant. For example, Celiker et al^[23]^ showed that the intensity of pain in FM patients was correlated with the anxiety level and depression of the patients.

Similarly, AS was found to be higher in the patient group than in the control group, according to the ASI results. AS was defined as “an extreme fear of anxiety-related sensations and symptoms believed to have harmful physical and/or social consequences.”^[6]^ According to this model, processes called “anxiety expectation and AS” play a role in the basis of people’s instinct to avoid a fearful event or situation. People with high AS are prone to misinterpreting sudden onset, relatively severe, and unexplained physical symptoms of anxiety as dangerous, and they often tend to avoid it. In our study, there was a significant difference with the control group only in terms of cognitive symptoms, among the three sub-headings of ASI, but no difference was found in terms of physical and social symptoms.

When the correlation between the quality of life evaluated with SF 36 and the results of the ASI-3 test is examined, the quality of life in terms of energy, mental health, general health, and physical function decreases as the physical symptoms, cognitive symptoms, and sensitivity to social symptoms increase in the patient group in terms of ASI-3. In addition to these partnerships, it was determined that as the sensitivity to cognitive symptoms increased in the patient group, the quality of life in terms of emotional role difficulty, social functionality, and pain decreased. According to these results, while the dimensions of quality of life determined and reduced by physical and social symptoms are the same, cognitive symptoms cause a more comprehensive decrease in quality of life. Considering that the main difference between the patient and control groups in terms of ASI-3 is in cognitive symptoms, the importance of the second decrease in quality of life for FM patients is clear.

Pain is a subjective and unmeasurable symptom. Some pain assessment methods are used to compare and evaluate them numerically. Among these, the most frequently used one in clinical practice and the literature is the VAS, and VAS was found to be high in FM patients.^[24]^ In our study, VAS scores were found to be significantly higher in FM patients. The increase in pain level determined by VAS decreases the quality of life in terms of physical role difficulty, social functionality and pain, just like anxiety level. As a result, it can be said that anxiety, depression and perceived pain severity of the FM patient group impair their social functionality more than healthy individuals.

The limitations of our study include the fact that male patients were not included in the study, no clinical evaluation was made in terms of psychiatric comorbidities other than the scales, and the number of years of FM complaints during the study was not included in the evaluation.

## 5. Conclusion

In this study, it was determined that the functionality and quality of life of the patients diagnosed with FM decreased. An important contribution of the study to the literature is that it shows that the behavior of avoiding activity due to pain-induced fear exacerbates the pain and even contributes to its chronicity. In addition, it has been determined that anxiety, depression, and perceived pain intensity decrease social functionality and quality of life in common areas such as mental health, physical function, and emotional role difficulties in FM patients.

When the results of the relationship between AS and quality of life were evaluated, it was observed that while the dimensions of quality of life determined and reduced by physical and social symptoms were the same in the patient and control groups, cognitive symptoms caused a more comprehensive decrease in quality of life in patients. In terms of ASI-3, the main difference between the patient and control group is the cognitive symptoms and the second decrease in quality of life indicates the importance of the psychological component of the disease.

Similar studies determining the effect of AS and fear avoidance behavior on the prognosis of the disease in FM patients are needed in larger samples and different cultures. All these results emphasize the importance of psychiatric evaluation and treatment in FM patients.

## Author contributions

**Conceptualization:** Sera Cetingok, Oznur Seker.

**Data curation:** Oznur Seker, Halil Cetingok.

**Formal analysis:** Sera Cetingok, Oznur Seker, Halil Cetingok.

**Methodology:** Sera Cetingok.

**Project administration:** Sera Cetingok, Oznur Seker, Halil Cetingok.

**Resources:** Oznur Seker, Halil Cetingok.

**Supervision:** Halil Cetingok.

**Validation:** Sera Cetingok, Halil Cetingok.

**Writing – original draft:** Sera Cetingok, Oznur Seker, Halil Cetingok.

**Writing – review & editing:** Sera Cetingok, Halil Cetingok.
